# Impact of Endourological procedures with or without double-J stent on sexual function: a systematic review and meta-analysis

**DOI:** 10.1186/s12894-020-0582-1

**Published:** 2020-02-14

**Authors:** Junlin Lu, Yinghong Lu, Yang Xun, Fan Chen, Shaogang Wang, Shiyi Cao

**Affiliations:** 1 Department of Urology, Tongji Hospital, Tongji Medical College, Huazhong University of Science and Technology, Wuhan, People’s Republic of China; 2 School of Public Health, Tongji Medical College, Huazhong University of Science and Technology, Wuhan, People’s Republic of China

**Keywords:** Sexual dysfunction, Double-J stent, Ureteroscopy, Urolithiasis, Endourological procedures

## Abstract

**Background:**

Endourological procedures are widely used to treat benign urinary disorders and the double-J stent is routinely used. However, its potential impact on sexual function remains unclear. Therefore, we performed a quantitative systematic review to determine the relationship between endourological procedures with or without double-J stent and post-operative sexual function.

**Methods:**

We conducted a search of PubMed, EMBASE, Web of Science, and Cochrane Library databases up to December 2018 for studies that compared sexual function before and after endourological procedures. The quality of the included studies was evaluated using the Risk Of Bias In Non-randomized Studies of Interventions (ROBINS-I). We performed subgroup analyses to explore heterogeneity. A random effects model was used to combine the results.

**Results:**

Five prospective studies involving 485 sexually active participants were identified. Pooled results showed that, in patients without a double-J stent, the change in sexual function after endourological procedures was not significant in men (mean difference [MD]: − 0.61, 95% confidence interval [CI]: − 1.43 to 0.22, *p* = 0.148) or women (MD: 0.53, 95% CI: − 0.52 to 1.57, *p* = 0.322). However, in patients with indwelling double-J stent, sexual function scores significantly declined after the procedure in both men (MD: -4.25, 95% CI: − 6.20 to − 2.30, *p* < 0.001) and women (MD: -7.17, 95% CI: − 7.88 to − 6.47, *p* < 0.001).

**Conclusions:**

Our meta-analysis suggests that indwelling double-J stent after endourological procedures could be a crucial factor causing temporary sexual dysfunction post-operatively. Our results may be used to provide evidence-based advice to patients.

## Background

Urinary stone is a common disease with a prevalence rate varying from 1 to 20% [1]. Endourological procedures (EP), including percutaneous nephrolithotomy (PCNL) and ureteroscopy (URS), are strongly recommended to remove stones according to guidelines [1, 2]. However, these transurethral procedures may have adverse effects on sexual function [3]. In 2003, Joshi et al. firstly found that indwelling double-J stent resulted in sexual problems in 35% of sexually active patients [4, 5]. Increasing concerns were raised about the impact of indwelling double-J stent or EP on sexual function. Sofer et al. utilized a validated questionnaire, the International Index of Erectile Function (IIEF) including five evaluating items, and demonstrated sexual dysfunction after EP regardless of whether double-J stent was applied [3]. It posed the question-is it double-J stent or EP itself that affected sexual function? With accumulated evidence, a pooled analysis is required to address the issue.

Sexual function depends on several functional domains. The International Consultation on Sexual Medicine (ICSM) recommended IIEF and Female Sexual Function Index (FSFI) as sexual dysfunction questionnaire for general population with level of evidence 1A [6]. They assess functions by different sexual feature in both sexes. The IIEF questionnaire for men includes five items: erectile function, orgasmic function, sexual desire, intercourse satisfaction and overall satisfaction; while the female FSFI questionnaire includes five items: desire, arousal, lubrication, orgasm, satisfaction and pain [7, 8]. The two questionnaires were widely used to evaluate sexual function related to surgeries like abdominal aortic aneurysm surgical repair and bariatric surgery [9, 10]. To address sexual dysfunction after EP, IIEF and FSFI are also the optimal tools.

Sexual activity and fulfillment are essential to maintain emotional well-being and quality of life [11]. Therefore, we conducted the first systematic review to assess the impact of the EP and double-J stent on sexual health, and to provide information for patients counseling when endourological interventions are under consideration. This result is also helpful for urologists to have a comprehensive understanding of the complications of transurethral surgery. Accordingly, optimized surgical strategy could be developed to reduce complications and promote fast recovery after surgery.

## Methods

### Search strategy

This systematic review was conducted in accordance with the Preferred Reporting Items for Systematic Review and Meta-analysis (PRISMA) guidelines [12]. Search was conducted in December 2018 without limitation of starting time of the literature, using PubMed, EMBASE, Web of Science, and Cochrane Library databases. The search keywords were as follows: (endourology OR ureteroscopy OR ureterorenoscopy OR percutaneous nephrolithotomy OR retrograde intrarenal surgery OR stent OR stenting OR stents) AND (sexual function OR sexual dysfunction OR international index of erectile function OR female sexual function index). Furthermore, we scrutinized the reference lists to identify further pertinent studies. The language was restricted to English.

### Study selection criteria

Studies meeting the following criteria were included: (1) the study design was prospective; (2) patients were diagnosed with benign urinary disorders including urolithiasis and benign ureteral obstruction; (3) the interventions were endourological procedures (mainly URS and PCNL) with or without ureteral stent; and (4) the outcome was alteration in sexual function assessed by IIEF or FSFI questionnaires. We excluded conference abstracts and studies with missing data. In addition, studies were excluded if sexually inactive patients were recruited.

### Quality assessment and data extraction

Two authors (J. Lu and Y. Lu) independently extracted information from the included studies and ranked the study quality based on the Risk Of Bias In Non-randomized Studies of Interventions (ROBINS-I) [13]. Inconsistencies were resolved by discussion with a third reviewer (S. Cao). Extracted data contained study information (first author, publication year, study design), participant characteristics (number of patients, sex, age, type of disease), intervention characteristics (type of endourological procedure, application of stent), outcome characteristics (questionnaires, score of sexual function, evaluation time). The ROBINS-I includes three categories: pre-intervention (confounding, selection of participants), intervention (classification of interventions), and post-intervention (deviations from intended interventions, missing data, measurements of outcomes and selection of the reported results). Each category is assessed as “low risk of bias”, “moderate risk of bias”, “serious risk of bias”, “critical risk of bias”, and “no information”.

### Statistical analysis

Combined mean difference with 95% confidence intervals was considered the effect size in this meta-analysis. We compared sexual function scores before and after surgery to assess the alteration. A random effects model was applied to pool the effect sizes. We used the I^2^ statistic to measure heterogeneity. Values of 0–30% represented minimal heterogeneity, 31–50% represented moderate heterogeneity, and > 50% represented substantial heterogeneity [14]. All statistical analyses were performed using STATA.11.0 (Stata Corp, College Station, Texas, USA). All tests were two-sided with a significance level of 0.05.

## Results

### Literature search

**Fig. 1:**
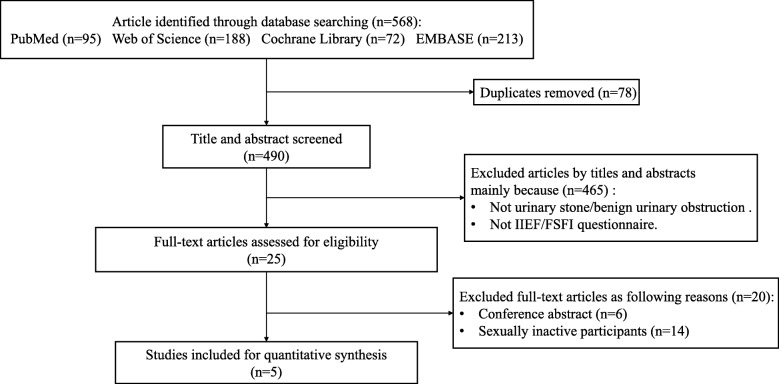
Flow chart of study search

The initial literature search identified total 568 articles: 95 from PubMed, 188 from Web of Science, 72 from Cochrane Library, and 213 from EMBASE. After removing 78 duplicates, we identified 490 citations. Through screening the titles and abstracts for eligibility, we retrieved 25 articles for full-text assessment. Of these, 20 articles were excluded because 14 articles recruited sexually inactive patients and six articles were conference abstracts. Finally, we included five prospective studies for meta-analysis. The research process is shown in Fig. 1.

### Characteristics and quality of the included studies

Table 1 shows the main characteristics of the five included studies. A total of 485 participants were sexually active. The study publication years ranged from 2007 to 2017. Studies were from three countries (one from Italy [15], one from Egypt [16] and three from Turkey [17–19]). All five studies were non-randomized controlled trials. Four studies were ranked as having a “moderate risk of bias” and one study was ranked as having a “serious risk of bias”. Detailed quality assessment by ROBINS-I is available in the Additional file 1. Table 1 lists the characteristics of EP including types of endourology, diameter of endoscope, and description of the stent (diameter, material, and length).

**Table 1:** The characteristics of included studies

Author (year)	Participants	Gender, age	Urinary disease	Endourological Procedures (diameter)	Stent			Questionnaires for sexual function	Time of evaluation		ROBINS-I	Reference
					Diameter	Material	Length		Cases with Stent	Cases without Stent		
Sighinolfi et al. (2007) [15]	50	Male,34–55 Female,28–51	Ureteral stones, retroperitoneal fibrosis	Endoscopy (NM)	4.7Fr or 6Fr	silicone and polyurethane	24 to 28 cm	(1) IIEF-5 (2) FSFI	①Pre-Pro^a^ ②Post-Pro^b^ (the 45th -60th day)		S	15
Mosharafa et al. (2016) [16]	100	Male,24–60	Urologic disorders	Ureteroscopy, PCNL, retrograde ureteropyelography, cystolitholapaxy (NM)	NM			(1) IIEF-5	①Pre-Pro ②Post-StR^c^ (the 10th day) ③Post-StR (the 45th day)	①Pre-Pro ②Post-Pro (the 10th day) ③Post-Pro (the 45th day)	M	16
Eryildirim et al. (2015) [17]	102	Male,27–64 Female,26–65	Ureteral stones	Ureteroscopy (semi-rigid,8Fr)	Non-stent			(1) IIEF (2) FSFI	①Pre-Pro ②Post-Pro (the 4th week)		M	17
Eryildirim et al. (2011)	177	Male and female,43.22 ± 1.52	Distal ureteral stones	Ureterorenoscopy (NM)	4.7Fr	polyuretan	26 cm or 28 cm	(1) IIEF (2) FSFI	①Pre-Pro ②Post-Pro (the 4th week)		M	18
Akdeniz et al. (2017) [18]	56	Female,22–58	Ureteral stones	Ureteroscopy, cystoscopy (rigid,7.5Fr)	NM			(1) FSFI	①Pre-Pro ②Post-StR (the 1st month) ③Post-StR (the 3rd month)	①Pre-Pro ②Post-Pro (the 1st month) ③Post-Pro (the 3rd month)	M	19

*Abbreviation: NM* not mentioned, *S* serious, *M* moderate

^a^Pre-Pro:Pre-procedure;

^b^Post-Pro: Post-procedure

^c^Post-StR: Post-Stent removal

### Results of meta-analysis

#### Relationship between sexual function and endourological procedures

**Fig. 2:**
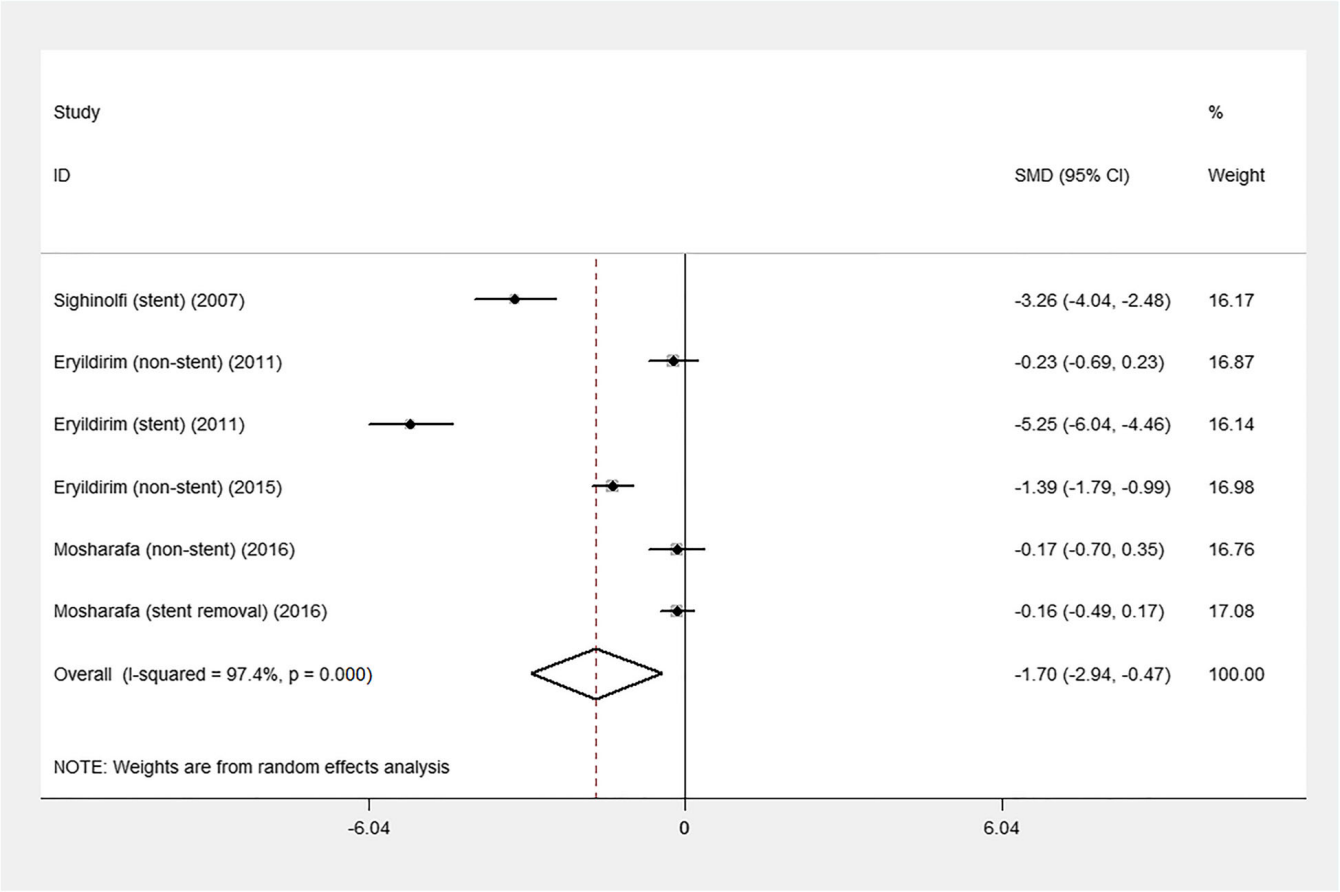
Forest plot of sexual function changes in men. CI, confidence interval

**Fig. 3:**
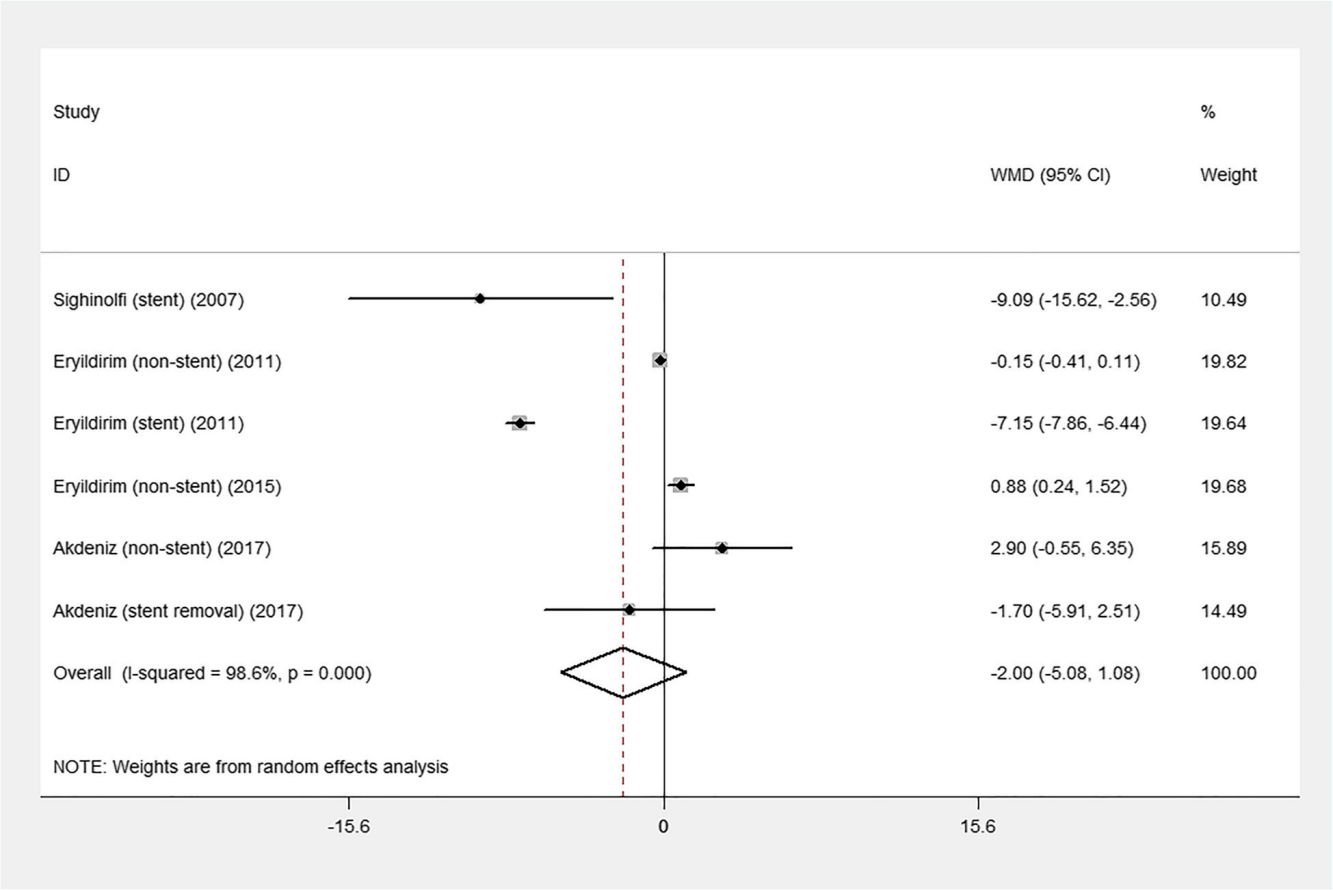
Forest plot of sexual function changes in women. CI, confidence interval

Figures 2 and 3 illustrate the results of the pooled mean difference (MD) and 95% confidence interval (CI) of the changes in sexual function in males and females, respectively. Of the five included studies, six groups comprised male patients while the other six groups comprised female patients. The MD of sexual function in men was − 1.7 (95% CI: − 2.94 to − 0.47; *p* = 0.007), and in women it was − 2.00 (95% CI: − 5.08 to 1.08; *p* = 0.204).

#### Subgroup analysis

The results of the subgroup analysis are shown in Table 2. We conducted subgroup analysis according to the stent-related state: with stent, without stent, and after stent removal. Stent group showed a significant decrease in sexual function in both sexes: in men, MD = − 4.25 (95% CI: − 6.20 to − 2.30; *p* < 0.001), and in women, MD = − 7.17 (95% CI: − 7.88 to − 6.47; *p* < 0.001).

**Table 2:** Results of subgroup analysis about endourological procedures and sexual function

Gender	Subgroup	Number of studies	MD	95% CI	*p* value	Study heterogeneity			
						χ^**2**^	df	p	I^2^
male	Stent	2	-4.25	−6.20 to −2.30	<0.001	12.28	1	<0.001	91.9%
	Non-stent	3	−0.61	−1.43 to 0.22	0.148	19.22	2	<0.001	89.6%
	Stent removal	1	−0.61	−0.49 to 0.17	0.341	–	–	–	–
female	Stent	2	−7.17	−7.88 to −6.47	<0.001	0.33	1	0.563	0.0%
	Non-stent	3	0.53	−0.52 to 1.57	0.322	11.24	2	0.004	82.2%
	Stent removal	1	−1.70	−5.91 to 2.51	0.429	–	–	–	–

*MD* mean difference.

*CI* confidence interval.

#### Subdomains of IIEF and FSFI

There are five domains in the IIEF questionnaire: erectile function (EF), intercourse satisfaction (IS), orgasmic function (OF), sexual desire (SD) and overall satisfaction (OS). In our study, two domains of IIEF were slightly impaired after the non-stent procedure: IIEF-EF (MD = − 1.34, 95% CI: − 2.41 to − 0.28; *p* = 0.014) and IIEF-OF (MD = − 0.58, 95% CI: − 0.77 to − 0.38; *p* < 0.001).

There are six domains in the FSFI questionnaire: sexual desire (SD), sexual arousal (SA), sexual lubrication (SL), sexual orgasm (SO), sexual satisfaction (SS), sexual pain (SP). In our study, only FSFI-SD was marginally impaired after the non-stent procedure in women (MD = − 0.06, 95% CI: − 0.11 to − 0.01; *p* = 0.013).

These results are shown in the Additional file 1.

## Discussion

Based on current evidence, the indwelling double-J stent negatively correlated with sexual function in all domains. Non-stent procedures had no impact on general sexual function in both sexes even though one or two domains of the administered questionnaires were mildly impaired, including erectile function and orgasmic function in men and sexual desire in women. One study reported that sexual deterioration in women recovered 1 month after stent removal [19]. In another study, the IIEF score remained unchanged on the 10th day after stent removal when compared with the preoperative baseline value [16]. These results suggest that sexual function was impaired after employing a stent but recovered soon following stent removal.

Sexual activity is an important aspect of individual life. The World Health Organization (WHO) describes sexual health as “a state of physical, emotional, mental, and social well-being in relation to sexuality.” [20] Moreover, sexuality is also a way of experiencing pleasure and physical release, facilitating quality of life and longevity [21]. Regular sexual activity varies from 71.3% in old people to 96% in young people. However, the prevalence of erectile dysfunction (ED) ranges from 2.3 to 53.4% according to age [22]. Women appear to be more vulnerable to sexual problems. As many as 40–45% of women have experienced sexual dysfunction during their lifetimes [23]. Unexpected deterioration of sexual function has a significant impact on patients after having undergone certain surgeries. Ureteroscopy preoperatively increases anxiety in patients because of its surgical approach. The passage of rigid material, urethral expansion, and stent placement are new and fear-inducing events for some patients. Patients frequently inquire about the effect these procedures have on sexual function. However, urologists often only provide a simple explanation in accordance with their clinical experience. This systematic literature review provides more accurate evidence for preoperative patient consulting.

Factors that increase the rate of sexual dysfunction after EP are quite complex. EP primarily refers to ureteroscopy, cystoscopy, and percutaneous nephrolithotomy. Upper urinary stones and urinary strictures are considered for EP because malignant diseases such as prostate cancer are often accompanied by nerve injury after radiotherapy or surgery [24]. Surgical injury from ureteroscopy does not directly involve nerves; however, bladder or urethral mucosa may be irritated during the procedure. This depends on the nature of the ureteroscope, its diameter, and rigidity. Two included studies reported detailed information regarding the endoscope. One utilized an 8-Fr semi-rigid ureteroscope, and the other used a 7.5-Fr rigid ureteroscope or cystoscope. No significant change in sexual function was found at the fourth week compared with that at the preoperative baseline. Patients were reported to have had a more comfortable experience after flexible ureteroscopy with decreased pain and anxiety [25]. Nevertheless, in general, sexual dysfunction rarely occurs, even after rigid endoscopy.

The double-J stent is used for drainage and to decrease the incidence of renal colic and ureteral obstruction. Stent-related complications follow the benefits, especially lower urinary tract symptoms (LUTS). A meta-analysis of RCTs compared stent and non-stent procedures to treat ureteral stones [26]. Dysuria, irritation, hematuria, urinary infection, and pain were the top five common complications. The stent group had the highest incidence of flank or voiding pain (31.4% stent vs. 19.7% non-stent), dysuria (51.2% stent vs. 23.0% non-stent), and irritation (45.0% stent vs. 24.2% non-stent). LUTS such as dysuria and irritation are strongly related to sexual dysfunction. A large population-based study reported that odds ratios for ED were 3.0 (storage LUTS vs. no LUTS), 2.6 (voiding LUTS vs. no LUTS) and 4.0 (combined LUTS vs. no LUTS) [27]. The results are similar to those of previous studies. Joshi et al. validated a ureteral stent symptom questionnaire (USSQ) that was widely used to assess stent-associated disturbances [4, 5]. The post-stent LUTS were urgency (57.5%), urge incontinence (18.5%), incomplete emptying (27.5%), and dysuria (18.5%). Two primary complications, LUTS and pain, led to secondary complications described in USSQ: worse general health, impaired work performance, and sexual problems. Apart from LUTS and pain, the evidence showed that the double-J stent also provoked unfavorable mental changes such as anxiety and depression [5, 28]. All stent-associated disturbances (LUTS, pain, mental disorder) may impair sexual health. Included studies in our review utilized specific questionnaires (IIEF and FSFI) to evaluate sexual function. We found a significant decrease of sexual function in stent patients. The parameters of the stent (rigidity, diameter, material, and length) may also affect the degree of complications. Nevertheless, to our knowledge, current studies have not concentrated on whether and how stent technology affects sexual function.

USSQ is utilized to assess the safety of a new stent or medications that can relieve symptoms after placement of a stent. Zhu et al. and Giannarini et al. showed impairment in sexual health in patients compared to that in healthy individuals at four weeks after stent placement [29, 30]. By contrast, some studies showed no significant difference when comparing sexual health at the fourth week after placement with that at the fourth week after removal [30, 31]. A slight improvement of symptoms after stent removal may account for these results. The authors concluded that patients tended to suffer sharp declines in sexual function after stent insertion and sexual function slowly recovered with indwelling time. After the stents were removed, sexual function returned to that at baseline. In addition, medications such as α-blockers and antimuscarinics are effective in relieving stent-related LUTS, pain, and sexual problems [32, 33].

There are several limitations about the systematic review. The primary limitation is the small number of eligible studies and sample sizes. The ROBINS-I tool shows significant bias for the studies. In addition, the sexual function assessment questionnaires were various and the heterogeneity was high in the male groups. Addressing the effect of EP or stent on sexual function still demands well-designed and large sample studies with a standard assessment tool.

## Conclusions

Based on current studies, non-stent EP is not likely to influence post-operative sexual function. Employing a double-J stent after surgery may cause temporary sexual dysfunction. This is the first systematic review regarding sexual function change after EP. Studies are lacking to provide definite answer. Further study could focus on stent-related complications including temporary or persistent sexual dysfunction. Better stent design of materials and coatings is encouraged. Moreover, stent use requires strict indications to avoid improper use and to promote fast recovery after surgery.

## Supplementary information

**Additional file 1.** Supplementary materials are available.

## Data Availability

The datasets in this work is available from the corresponding author.

## Abbreviations

CI: Confidence interval
ED: Erectile dysfunction
EP: Endourological procedures
FSFI: Female Sexual Function Index
IIEF: International Index of Erectile Function
LUTS: Lower urinary tract symptoms
MD: Mean difference
PCNL: Percutaneous nephrolithotomy
URS: Ureteroscopy
USSQ: Ureteral stent symptom questionnaire
