# Supplementary material for: Impact of Endourological procedures with or without double-J stent on sexual function: a systematic review and meta-analysis
Source: BMC Urol. 2020 Feb 14;20:13. doi: 10.1186/s12894-020-0582-1 (PMC7023811; doi:10.1186/s12894-020-0582-1)

**Supplementary materials**

Table 1: Results of the assessment of risk of bias in included studies

Figure legends:

Figure 1: Forest plots of International Index of Erectile Function (IIEF) in non-stent patients. (A) Erectile function, (B) Intercourse satisfaction, (C) Orgasmic function, (D) Sexual desire, (E) Overall satisfaction.

Figure 2: Forest plots of Female Sexual Function Index in non-stent patients. (A) Arousal, (B) Desire, (C) Lubrication, (D) Orgasm, (E) Satisfaction, (F) Pain

Table 1: Results of the assessment of risk of bias in included studies

| Study ID | 1. Bias caused by confounding | 2. Bias caused by  selection of  participants | 3. Bias caused by  classification of  interventions | 4. Bias caused by  deviations from  intended  interventions | 5. Attrition bias  caused by  missing data | 6. Detection bias  caused by  measurement of  outcomes | 7. Reporting bias  caused by  selection of the  reported results | Overall  judgement |
| --- | --- | --- | --- | --- | --- | --- | --- | --- |
| Sighinolfi et al. (2007) | MODERATE | MODERATE | SERIOUS | LOW | MODERATE | MODERATE | LOW | SERIOUS |
| Mosharafa et al. (2016) | MODERATE | SERIOUS | MODERATE | LOW | LOW | MODERATE | LOW | MODERATE |
| Eryildirim et al. (2015) | MODERATE | MODERATE | SERIOUS | LOW | LOW | MODERATE | LOW | MODERATE |
| Eryildirim et al. (2011) | MODERATE | SERIOUS | MODERATE | LOW | LOW | MODERATE | LOW | MODERATE |
| Akdeniz et al. (2017) | MODERATE | SERIOUS | MODERATE | LOW | LOW | MODERATE | LOW | MODERATE |

Figure 1: Forest plots of International Index of Erectile Function (IIEF) in non-stent patients. (A) Erectile function, (B) Intercourse satisfaction, (C) Orgasmic function, (D) Sexual desire, (E) Overall satisfaction.

A. IIEF-Erectile function


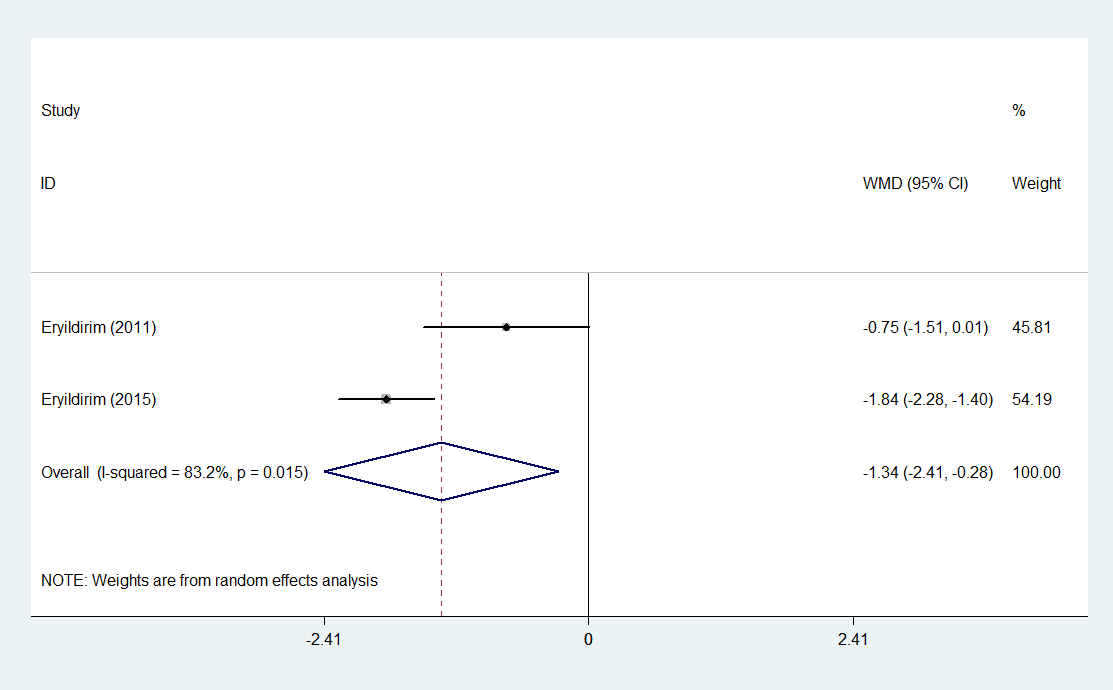


B. IIEF-Intercourse satisfaction


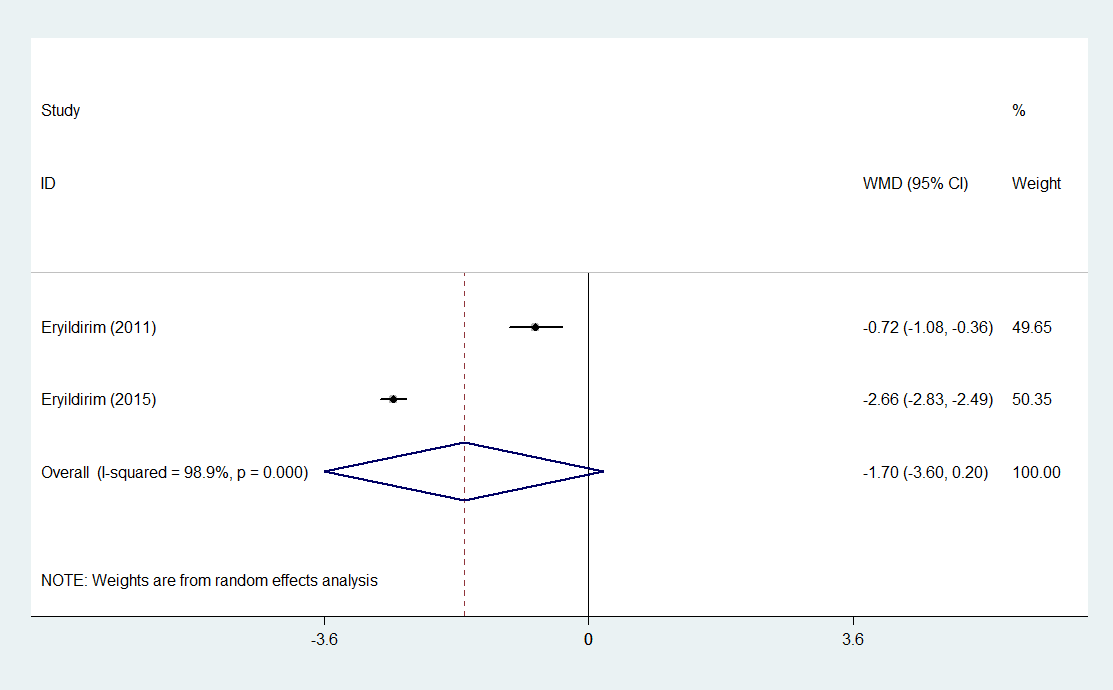


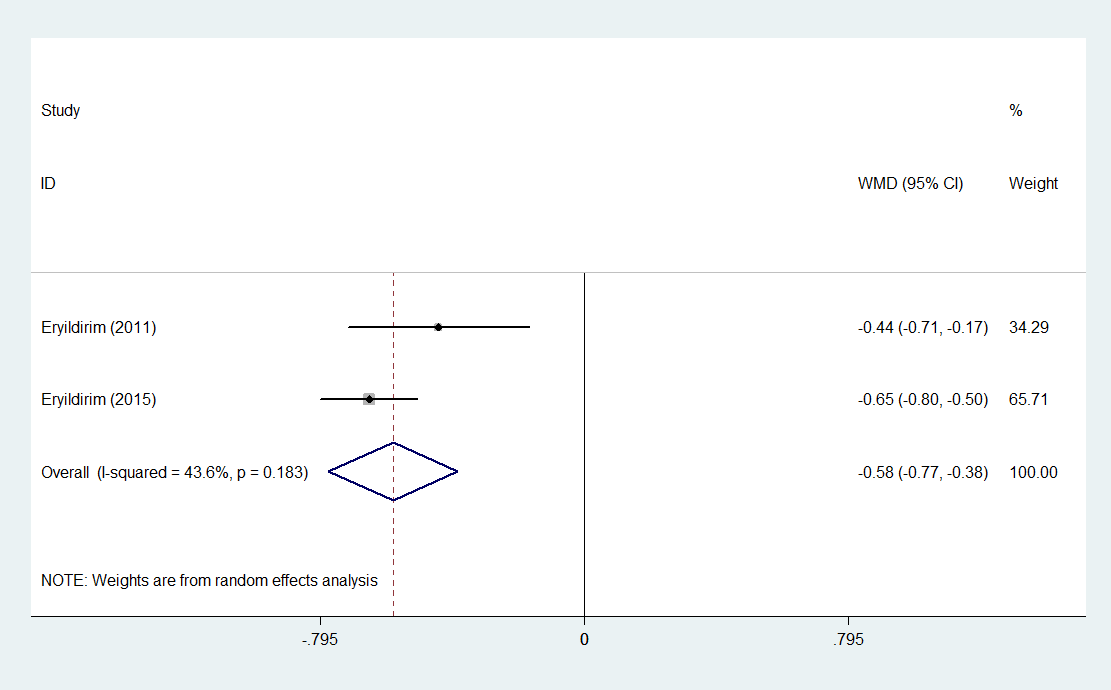
C. IIEF-Orgasmic function


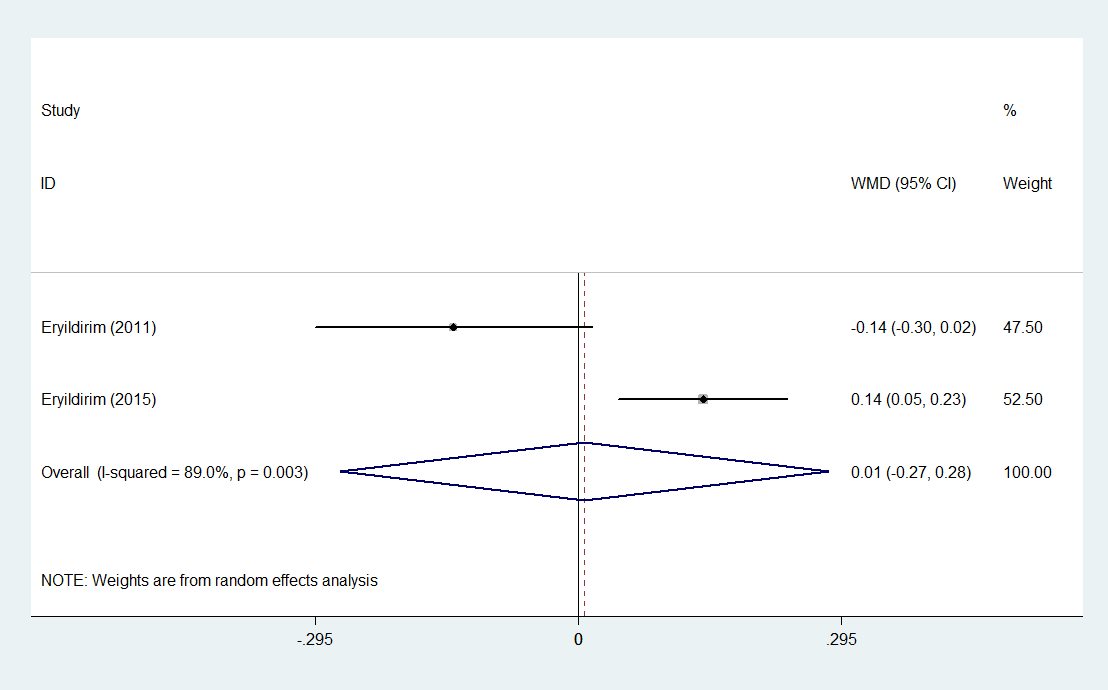
D. Sexual desire

E. Overall satisfaction


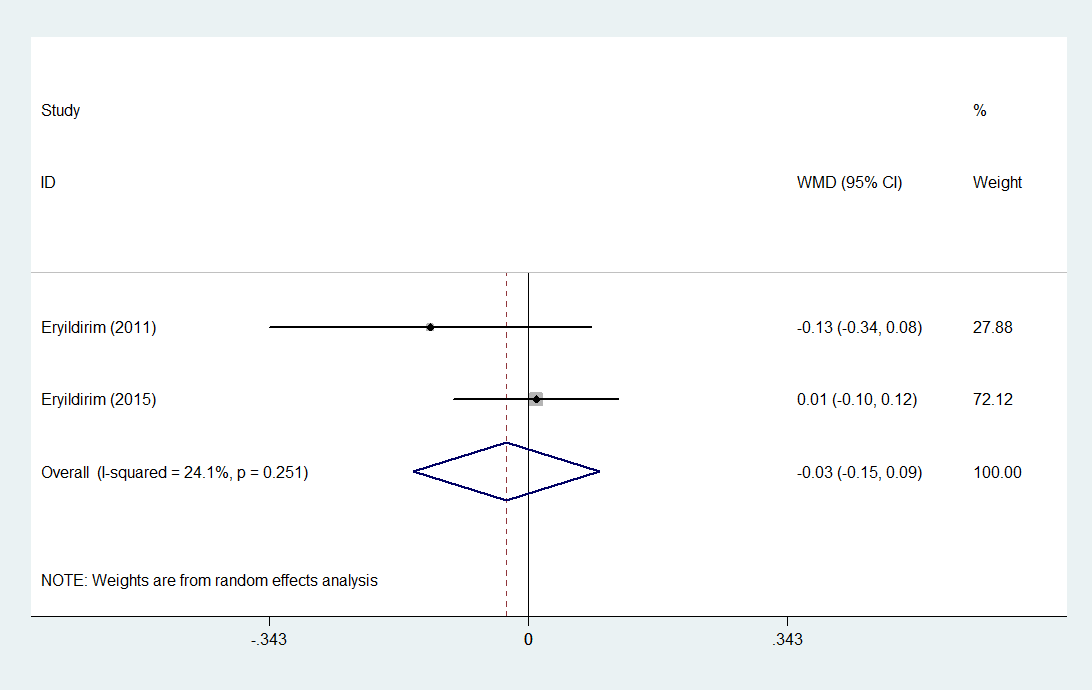


Figure 2: Forest plots of Female Sexual Function Index in non-stent patients. (A) Arousal, (B) Desire, (C) Lubrication, (D) Orgasm, (E) Satisfaction, (F) Pain.


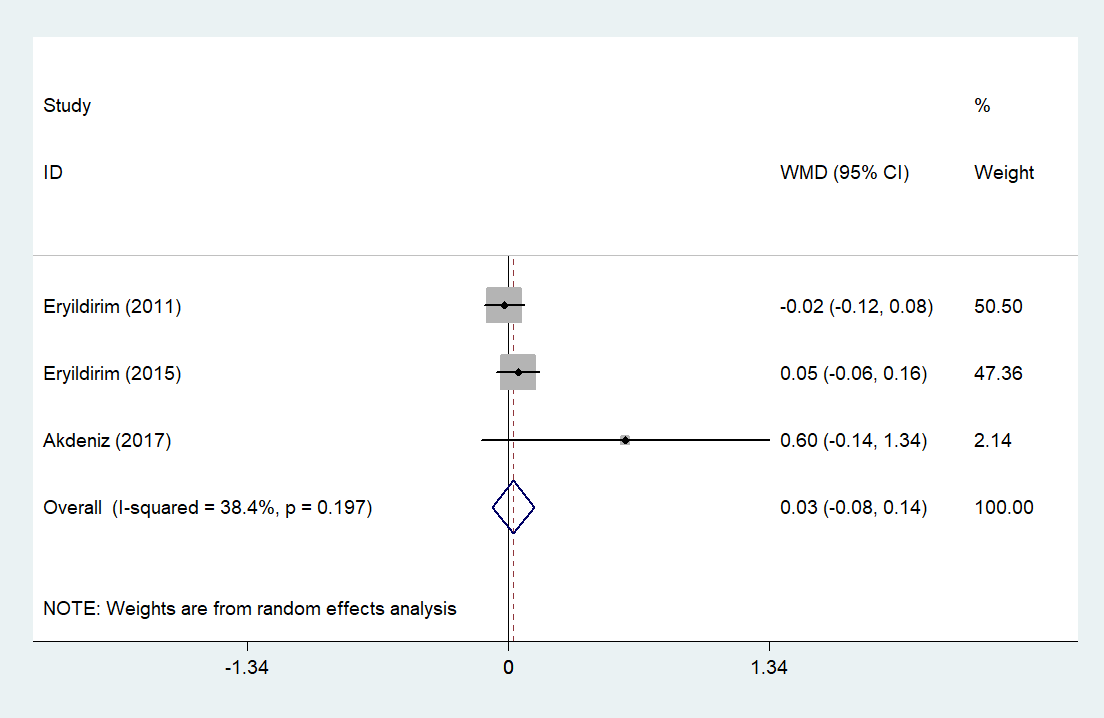
A. FSFI-Arousal


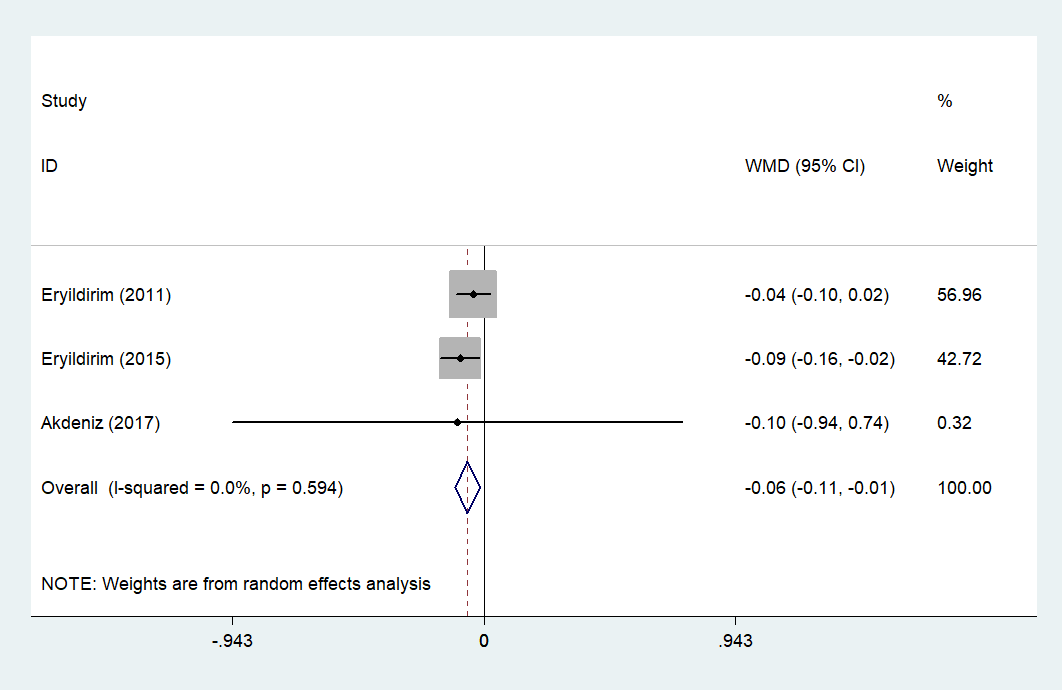
B. FSFI-Desire


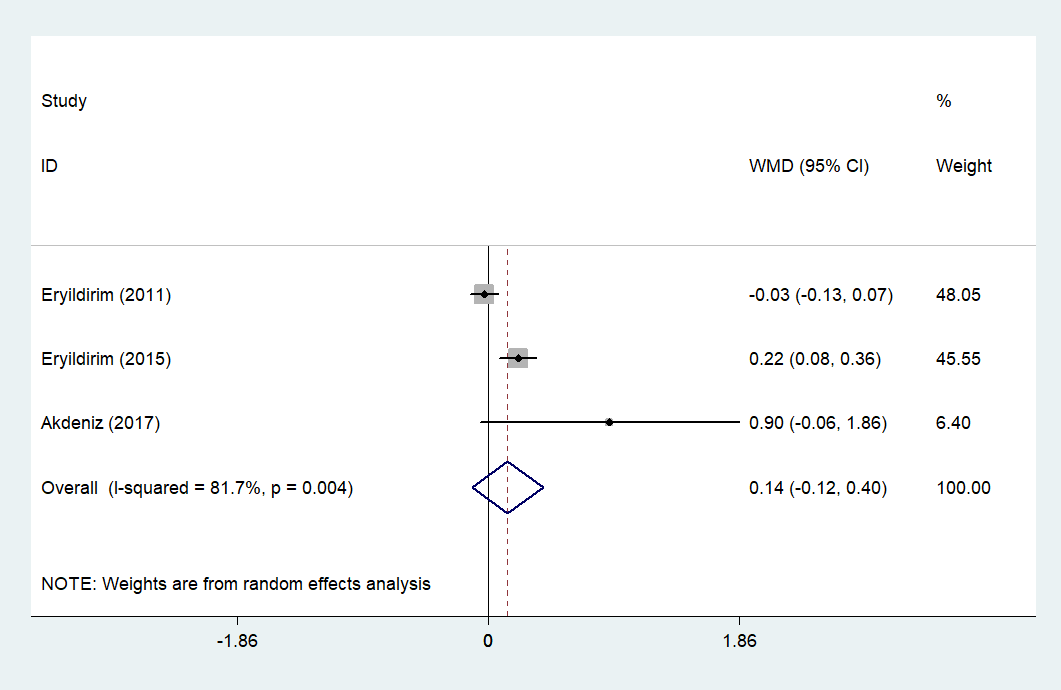
C. FSFI-Lubrication


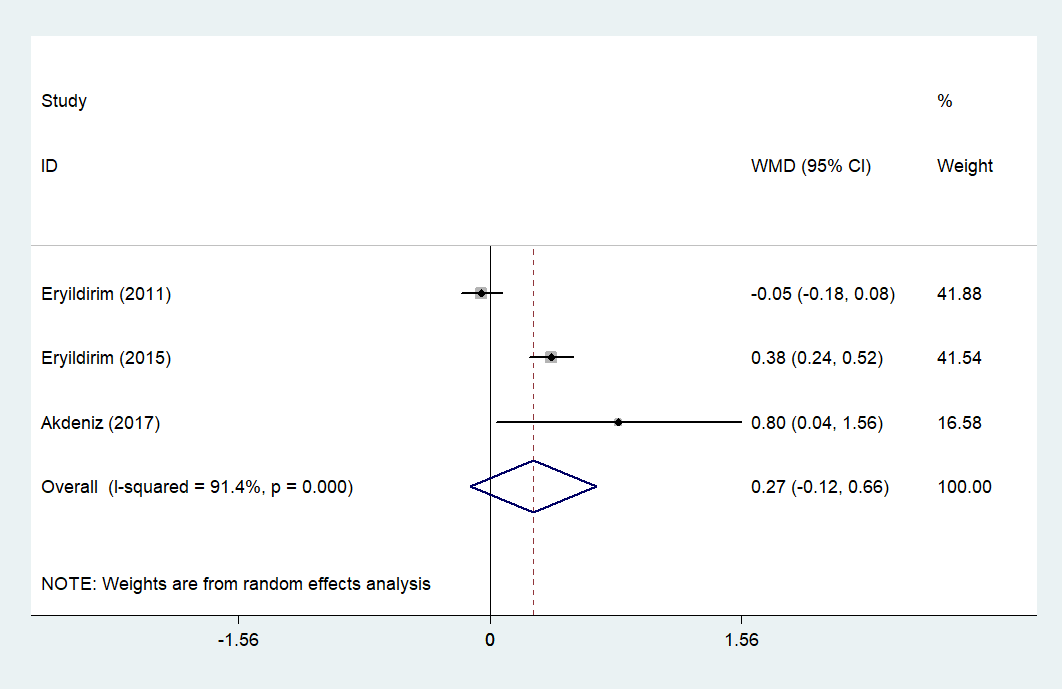
D. FSFI-Orgasm


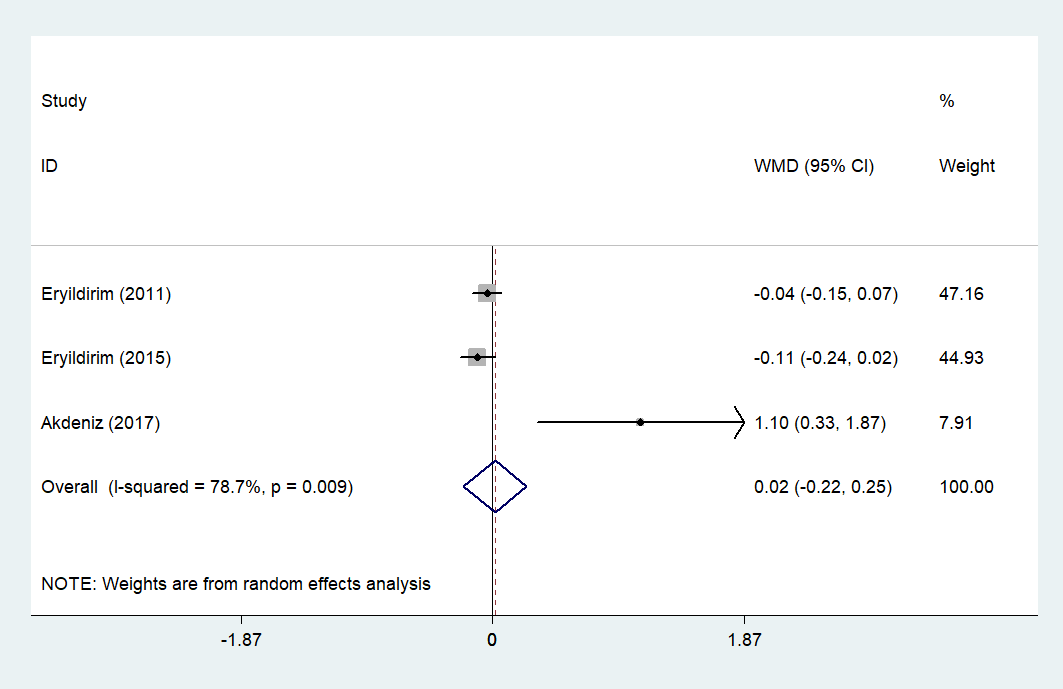
E. FSFI-Satisfaction

F. FSFI-Pain


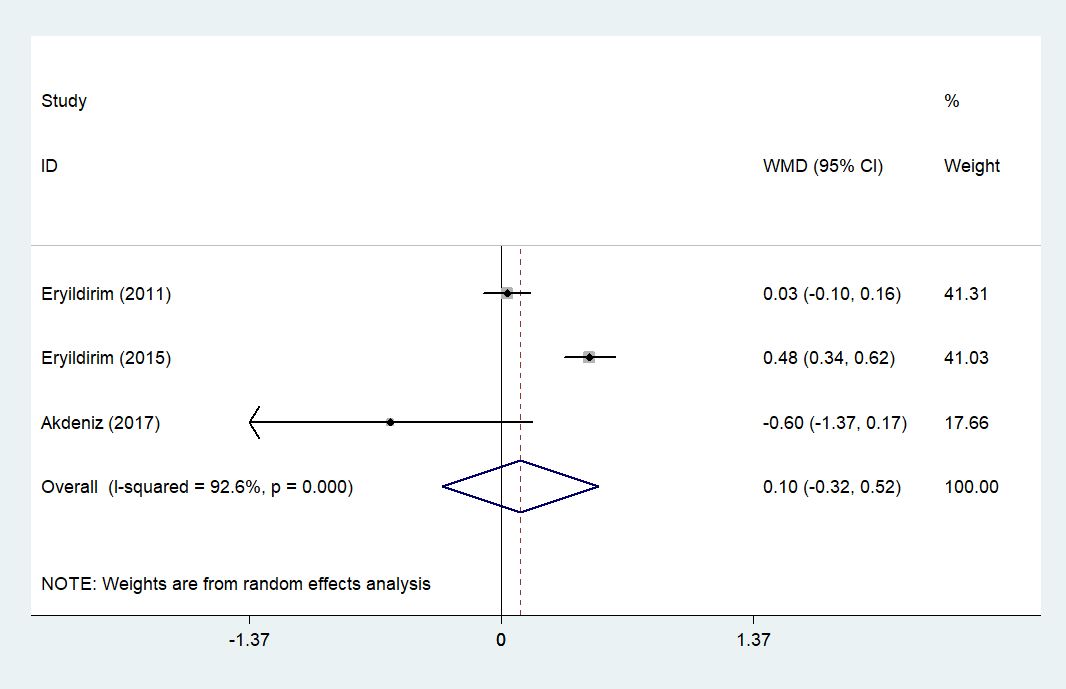

Supplement: Supplementary file 1 — Additional file 1. Supplementary materials are available. [file 12894_2020_582_MOESM1_ESM.docx]
